# Influence of Human Factors on Cyber Security within Healthcare Organisations: A Systematic Review

**DOI:** 10.3390/s21155119

**Published:** 2021-07-28

**Authors:** Sokratis Nifakos, Krishna Chandramouli, Charoula Konstantina Nikolaou, Panagiotis Papachristou, Sabine Koch, Emmanouil Panaousis, Stefano Bonacina

**Affiliations:** 1 Department of Learning, Informatics, Management and Ethics, Karolinska Institutet, 171 77 Solna, Sweden; panagiotis.papachristou@ki.se (P.P.); sabine.koch@ki.se (S.K.); stefano.bonacina@ki.se (S.B.); 2 School of Electronic Engineering and Computer Science, Queen Mary University of London, London E1 4NS, UK; krishna.chandramouli@qmul.ac.uk; 3 Natural Resources Institute, University of Greenwich, Kent ME4 4TB, UK; c.k.nikolaou@greenwich.ac.uk; 4 School of Computing and Mathematical Sciences, University of Greenwich, London SE10 9LS, UK; e.panaousis@greenwich.ac.uk

**Keywords:** cybersecurity, social engineering attacks, human factors, cyber risk assessment, cybersecurity awareness training

## Abstract

*Background:* Cybersecurity is increasingly becoming a prominent concern among healthcare providers in adopting digital technologies for improving the quality of care delivered to patients. The recent reports on cyber attacks, such as ransomware and WannaCry, have brought to life the destructive nature of such attacks upon healthcare. In complement to cyberattacks, which have been targeted against the vulnerabilities of information technology (IT) infrastructures, a new form of cyber attack aims to exploit human vulnerabilities; such attacks are categorised as social engineering attacks. Following an increase in the frequency and ingenuity of attacks launched against hospitals and clinical environments with the intention of causing service disruption, there is a strong need to study the level of awareness programmes and training activities offered to the staff by healthcare organisations. *Objective:* The objective of this systematic review is to identify commonly encountered factors that cybersecurity postures of a healthcare organisation, resulting from the ignorance of cyber threat to healthcare. The systematic review aims to consolidate the current literature being reported upon human behaviour resulting in security gaps that mitigate the cyber defence strategy adopted by healthcare organisations. Additionally, the paper also reviews the organisational risk assessment methodology implemented and the policies being adopted to strengthen cybersecurity. *Methods:* The topic of cybersecurity within healthcare and the clinical environment has attracted the interest of several researchers, resulting in a broad range of literature. The inclusion criteria for the articles in the review stem from the scope of the five research questions identified. To this end, we conducted seven search queries across three repositories, namely (i) PubMed^®^/MED-LINE; (ii) Cumulative Index to Nursing and Allied Health Literature (CINAHL); and (iii) Web of Science (WoS), using key words related to cybersecurity awareness, training, organisation risk assessment methodologies, policies and recommendations adopted as counter measures within health care. These were restricted to around the last 12 years. *Results:* A total of 70 articles were selected to be included in the review, which addresses the complexity of cybersecurity measures adopted within the healthcare and clinical environments. The articles included in the review highlight the evolving nature of cybersecurity threats stemming from exploiting IT infrastructures to more advanced attacks launched with the intent of exploiting human vulnerability. A steady increase in the literature on the threat of phishing attacks evidences the growing threat of social engineering attacks. As a countermeasure, through the review, we identified articles that provide methodologies resulting from case studies to promote cybersecurity awareness among stakeholders. The articles included highlight the need to adopt cyber hygiene practices among healthcare professionals while accessing social media platforms, which forms an ideal test bed for the attackers to gain insight into the life of healthcare professionals. Additionally, the review also includes articles that present strategies adopted by healthcare organisations in countering the impact of social engineering attacks. The evaluation of the cybersecurity risk assessment of an organisation is another key area of study reported in the literature that recommends the organisation of European and international standards in countering social engineering attacks. Lastly, the review includes articles reporting on national case studies with an overview of the economic and societal impact of service disruptions encountered due to cyberattacks. *Discussion:* One of the limitations of the review is the subjective ranking of the authors associated to the relevance of literature to each of the research questions identified. We also acknowledge the limited amount of literature that focuses on human factors of cybersecurity in health care in general; therefore, the search queries were formulated using well-established cybersecurity related topics categorised according to the threats, risk assessment and organisational strategies reported in the literature.

## 1. Introduction

Digital transformation, defined by Faddis [1], is a term used to describe the holistic effect created by a software application that fundamentally transforms a particular domain. In the historical context, digital transformation was adopted within the healthcare industry with examples including the system integration of health information systems and cybersecurity measures for networked medical devices [2]. However, with the ongoing COVID-19 pandemic, the adoption rate of such technologies has been expedited [3]. In [1], the authors stated that the responsibility of healthcare technology managementprofessionals is to offer expert consultation on the utilisation of healthcare facilities by clinical staff and overseeing the maintenance and operation of medical devices throughout their life cycle. The technical experts integrate health technologies, offering the ability to leverage medical technologies to provide better and safer patient care.

According to the technical series published by World Health Organisation (WHO) on primary health care [4] information and communication technology (ICT) is increasingly becoming common place with the introduction of smart phones, tablets and laptop computers. From technology that allows people to manage their health more effectively, better ways of diagnosing diseases, to monitoring the impact of policies on population health, digital technologies for health are influencing how health services are delivered and operated. One of the biggest barriers to the adoption of digital transformation strategies is cyber crime, which is responsible for exploiting the vulnerabilities of systems as well as human-attributed weakness. Cyber crime emerged in the late 1970s as the computer information technology (IT) industry took shape [5]. What began as *spam* eventually transitioned into computer viruses and malware (e.g., WannaCry). The health industry is an attractive target for cyber criminals, as health documents contain sensitive personal and financial information.

The rise of cybersecurity incidents is a growing threat to the healthcare industry, in general, and to hospitals in particular [6]. While the impact of cybersecurity is not unique to the healthcare industry, concerted efforts in protecting the stakeholder’s data has lagged behind and been lacking in healthcare in comparison to other industries [7]. A detailed list of various forms of cyber attacks is summarised in Table A1. With the fast digitisation of patient health records, the impact of data breaches on hospitals causes major economic and intangible damage [6]. To counteract the impact of such cybersecurity attacks, organisations have adopted governance strategies to promote best practices for securing the electronic infrastructure of hospitals and other clinical environments [6,8]. Despite the economic challenges often encountered by healthcare organisations in delivering healthcare services, there is increasing evidence of investment from hospital management to strengthen the ICT infrastructure [8]. While such a change might be voluntary, the data governance policies enacted by national authorities have also influenced the investment priorities.

However, the successful adoption of digital transformation strategies within healthcare industry relies on the successful acceptance among healthcare professionals towards addressing risks posed by cyber threats. Thus, it is important to deliver *awareness* and *training* programmes for healthcare professionals. The role of human behaviour in coping with cyberattacks and strengthening cyber defences is grouped into the theme of “human factors” in cybersecurity.

Following the increasing number of articles being published, addressing the role of humans in the loop for enhancing the cybersecurity since 2016 [8,9,10,11,12,13,14,15,16,17,18,19,20,21,22,23,24,25,26,27,28], there is a need to consolidate the research findings. The main objective of this systematic review is to review the literature to gather evidence from organisational case studies, author observations and scientific developments in cybersecurity to identify the role of including humans in the loop for strengthening cyber defence within the healthcare industry. To achieve this objective, in this systematic review, we review a detailed list of articles extending for more than a decade that address the challenge of cybersecurity when perceiving organisational threats and personal attacks of private information of a healthcare professional.

The contributions of the paper include the following:

- Conducting an extensive review of different types of cyber attacks encountered by healthcare and clinical environments that are reported in the literature.
- Studying the organisational defence strategies against cyber threats.
- Analysing the methodology adopted by different healthcare organisations for conducting cyber risk assessment.
- Evaluating the effect and impact of human factors in strengthening/weakening the cyber defence of a healthcare organisation.
- Reviewing national and international use cases on the impact of data breaches economically and the loss of intangible assets.

The rest of the paper is structured as follows. In Section 2, an overview of the Preferred Reporting Items for Systematic reviews and Meta-Analyses (PRISMA) [29] methodology adopted in the preparation of the systematic review is presented. Subsequently, the results analysis is presented in Section 3, which includes a detailed review of articles that address the different research questions as identified in Table 1. The opportunities for conducting additional research in the context of strengthening the cybersecurity defence of an organisation by investing in cybersecurity awareness activities and training programmes is summarised in Section 4. The conclusions and future work are summarised in Section 5.

## 2. Methodology

In this section, the step-by-step methodology conducted for the systematic review is presented. The methodology adopts the recommendations and guidelines provided in the PRISMA framework [29].

### 2.1. Research Questions

The scope of cybersecurity within healthcare is vast, ranging from technical developments to the organisational defence strategies adopted. Therefore, we have formulated the following five key research questions, which aim to categorise the literature across several topics as outlined in Table 1. Each of the research questions was formulated to identify the challenges often encountered by healthcare organisations in improving their cyber defence capabilities. A special emphasis is placed on those articles that consider the role of the healthcare staff (including executives, administrators, IT personnel, doctors, nurses, patients, etc.). To generate suitable responses for addressing these research questions, specific search strings were designed to search on bibliographic databases.

### 2.2. Protocol and Eligibility Criteria

We derived the structure of this systematic review from the PRISMA framework [29]. We considered articles to be eligible for this review if they were published in the last 11–12 years and the full-text version of the manuscript was published as Open Access. The selected articles were included only if they were peer-reviewed. As mentioned earlier, the scope of cybersecurity research topic in healthcare is a vast topic; in the time span under consideration, a few tens of thousands of articles have been published. Therefore, the eligibility criteria for a paper include the scope of study being within healthcare settings and clinical environment because organisations are responsible for storing patient records (e.g., electronic health records). The organisational resilience of hospitals and similar clinical environments were reviewed with a focus on one or more of the research questions outlined in Table 1.

### 2.3. Information Sources

**Figure 1:**
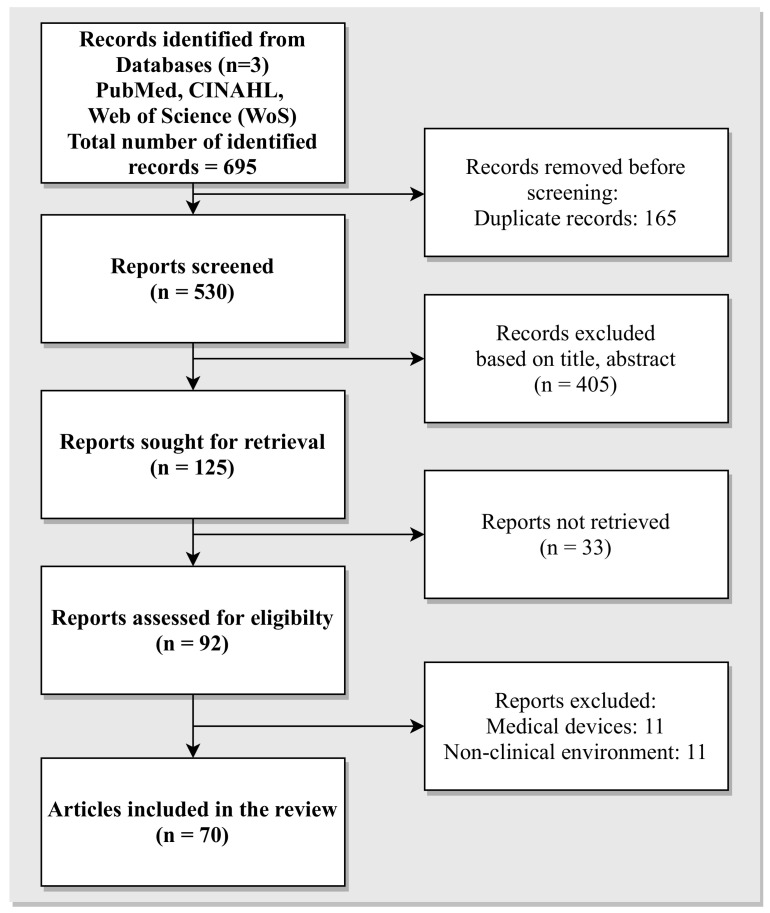
Literature search process, according to PRISMA recommendations.

A total of eight different search strings were used to search across three bibliographic databases, namely PubMed^®^/MEDLINE, Cumulative Index to Nursing and Allied Health Literature (CINAHL), and Web of Science (WoS). The search results from different databases were exported from the respective platform and further imported into the “Rayyan” [36,37] platform for collaborative filtering of the articles. Rayyan offers an online platform that supports a blind review of the articles by multiple authors. The imported results from PubMed, CINAHL, and WoS are individually analysed in accordance with the inclusion and exclusion criteria outlined in the previous subsection. With the “Blind On” feature, the results are not visible to other researchers, thus mitigating the risk of being biased. An overview of the literature search process in compliance to the recommendation provided by PRISMA is illustrated in Figure 1.

### 2.4. Searches

We conducted a total of eight queries of which queries 3 to 7 were executed both in PubMed and CINAHL, while query 8 was executed in the Web of Science (WoS) repository. The eight queries used to extract the results are the following:

1. Human[MeSH] AND (Cybersecurity[TIAB] AND “training” [TIAB] OR “Information security awareness” [TIAB]) AND 2010:2021[DP] AND eng[LA].
2. Humans[MeSH] AND (Cybersecurity[TIAB] OR “security awareness” [TIAB] OR “Information security awareness” [TIAB]) AND 2010:2021[DP] AND eng[LA].
3. cybersecurity AND (human[MeSH] AND (awareness AND healthcare)) AND 2010:2021[DP] AND eng[LA].
4. Healthcare[TIAB] AND (social engineering AND (Organisational policy AND (cybersecurity))) AND 2010:2021[DP] AND eng[LA].
5. Cybersecurity[TIAB] AND (social engineering OR (healthcare professionals)) AND 2010:2021[DP] AND eng[LA].
6. Cybersecurity[TIAB] AND (Healthcare[TIAB] OR (social engineering AND (training OR awareness))) AND 2010:2021[DP] AND eng[LA].
7. Healthcare AND (Cybersecurity[TIAB] AND (“training” [TIAB] OR “awareness” [TIAB])) AND 2010:2021[DP] AND eng[LA].
8. (TS = (Cybersecurity* AND Awareness AND Healthcare)) AND LANGUAGE: (English) AND DOCUMENT TYPES: (Article) Timespan: 2010–2021. Indexes: SCI-EXPANDED, SSCI, A&HCI, CPCI-S, CPCI-SSH, ESCI.

The results from the above search queries are varied. Query 1 resulted in 17 articles, while query 2 resulted in 217 articles. The query executed on WoS (query eight) resulted in 221 total search results. In summary, a total of 695 articles were retrieved. After the application of the filtering process outlined in Figure 1, a total of 70 papers were selected for this systematic review. The filtering process was carried out as recommended by the PRISMA guidelines.

### 2.5. Inclusion and Exclusion Criteria

We adopted a hierarchical application of inclusion/exclusion criteria in the filtering process. During the first stage filtering, it was unanimously agreed among the researchers that each article should be screened for generic criteria that establish the scope of the systematic review.

The following inclusion criteria were used to identify papers to be included:

- Articles that report on cyber threats/attacks aimed at hospitals and other clinical environments.
- Articles that identify vulnerabilities exploited by the relevant cyber attackers.
- Studies relevant to organisational cybersecurity risk assessment.
- Articles that report on national case studies with emphasis on cyber defence strategies.

The following exclusion criteria were used to screen for irrelevant primary studies:

- Studies not relevant to our research questions.
- Studies written in a language other than English.
- Duplicates and repeated studies.
- Articles that are not directly related to the hospitals and other clinical environments.
- Articles that report on cybersecurity of medical devices.
- Articles that report on patient safety from medical devices and/or relevant cybersecurity technologies.
- Studies that report only on technical development (e.g., algorithms, software) without the involvement of healthcare professionals.

These inclusion and exclusion criteria were summarised using three-stage classification: (i) include; (ii) exclude; and (iii) maybe. These three levels of categorisation were adopted to eliminate the articles that are not within the scope of the systematic review. Following the assignment of the include/exclude/maybe categories from each researcher, the articles listed in the “maybe" category were subsequently analysed. Each article categorised as “maybe” was subjected to further review by a minimum of two researchers and an unanimous agreement was reached to either include or exclude relevant articles. Using this process, 695 selected papers was reduced to 92 articles to be further analysed, which is outlined in the next subsection.

### 2.6. Article Selection

Following the selection of 92 articles, in the next round of iteration, a subjective ranking mechanism was agreed by the researchers to individually assign the relevance of each article to each of the five research questions identified in Section 2. It is also important to note that the individual scores assigned to each paper across each research question were subsequently normalised to a single measure by taking an average if the deviation was less than 5%. If the deviation was larger than 5%, then a consensus meeting was organised to agree upon the final score to be assigned to the relevant article. The rationale for choosing this approach is to quantify the overlap and interdependence of the relevant studies in addressing the topic of cybersecurity. Out of 92 articles, 20 articles were excluded, as they did not relate to the research questions. Finally, a total of 70 articles were selected that satisfy the criteria set forth for this systematic review.

### 2.7. Data Extraction

At every step of the systematic review process, all the information was stored in the Rayyan platform. Rayyan is an online collaborative platform that supports the filtering process carried out among the researchers. While there exist several tools to manage the collection of research articles, the use of the Rayyan platform resulted in an efficient and effective practice of reviewing each article collected for the review. The platform was used to store fulltext in “.PDF” (portable document format) for subsequent review. In addition, each paper was also annotated to indicate the relevance of this article to the research questions. Such annotated versions were individually stored by each researcher, which were used as evidence to back up the claims on the relevance. The discussions between the researchers were constructive and fed directly to improve the quality of the research. In general, discussions on the scale of the national pilot studies to be included were discussed in detail (RQ5), as opposed to any other research questions.

### 2.8. Risk of Bias

The topic of cybersecurity in health care has been subjected to several studies in the literature and thus, the researchers were tasked with establishing the exclusion criteria to limit the scope of the articles to be included in the study. The subjective ranking offered by individual researchers on the levels of relevance of each article against the different research questions was considered to introduce the risk of bias. To mitigate such a bias, the researchers conducted individual rankings and organised regular meetings to discuss the individual rankings.

## 3. Results

**Figure 2:**
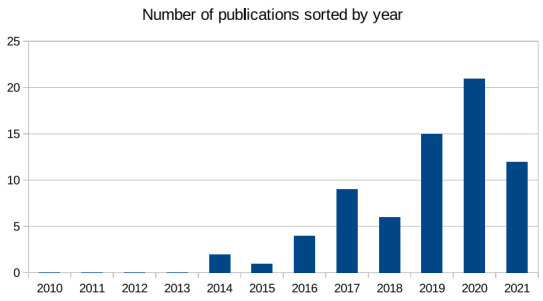
The selected 70 articles grouped according to the year of publication.

**Figure 3:**
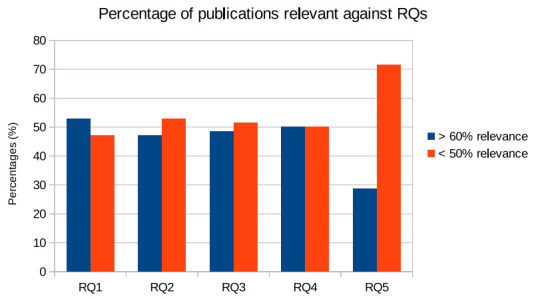
Relevance of articles addressed against each RQs, as outlined in Table 1.

**Figure 4:**
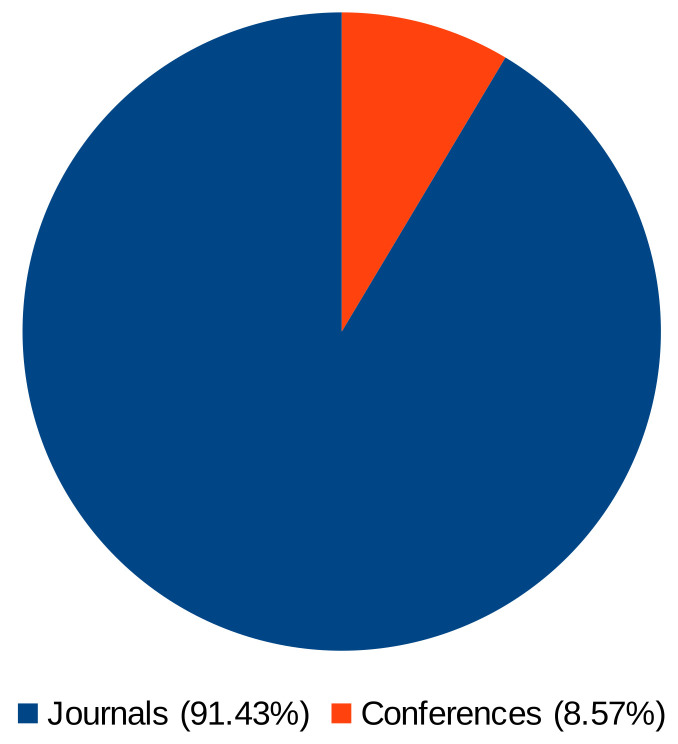
Distribution of article types for 70 publications (64 journal papers and 6 conference papers) included in the systematic review.

The search results for the 70 papers are presented in Figure 2, Figure 3 and Figure 4. As noted in Figure 2, the topic of cybersecurity in confluence with organisational awareness and training is a necessity. It is also evident that the frequency of such articles becoming published is gradually increasing year over year, even in the short span of six months in 2021 [3,7,17,22,23,24,26,34,38,39,40,41]. The total number of articles in this domain is already almost half the number of published articles from last year, and more than 75% of the articles from 2019. Similarly, the individual rankings associated to these articles and their relevance also provide a key insight into the challenges addressed by researchers, presented in Figure 3. In RQ1, we identified the nature of cybersecurity attacks being experienced by healthcare organisations, and the articles selected in the review received higher rankings than the papers with a lower ranking. This is due to the fact that several articles reported studies on the impact of ransomware (e.g., WannaCry) [20,32,42,43] and phishing attacks [9,10,13,14,15,41,44,45,46,47], to name a few being launched against healthcare organisations.

The percentage of articles addressing the organisational cyber resilience policies and governance is found to be less reported (as outlined in RQ2, [6,17,30,31]), along with the number of articles that address methodologies for healthcare organisations to conduct cybersecurity risk assessments (as presented in RQ3, [7,17,25,40,41,43,48,49]). The articles reporting on the training and awareness of cybersecurity among healthcare stakeholders are presented in the literature with equal distribution of relevance as highlighted in Figure 3 for RQ4 [8,9,10,11,12,13,14,15,16,17]. From the percentage results presented in Figure 3 for RQ5, it appears that there is a critical lack of national audits within the healthcare industry, which reports on the cyber resilience [8,9,10,22,31,50,51,52]. Among the total number of articles selected for this survey, 91.43% articles (64) are selected from peer-reviewed, high-impact journals, while only 8.57% articles (6) are selected from reputed conferences as presented in Figure 4. The consolidated rankings provided to each of the individual articles are presented in Table A2 of Appendix B. The scale of the matching score extends from 0, which represents no correlation of the publication to the corresponding research question, to 10 representing a high degree of correlation and relevance of the articles to the research question. As noted earlier, the interdependence between the research questions has necessitated the adoption of ranking metrics instead of opting for a binary assignment, e.g., yes/no to the relevance to each RQ.

### 3.1. Cyber Threats Commonly Encountered within Healthcare Organisations

The literature on cyber threats often encountered by healthcare organisation can be broadly classified into three main categories, namely (i) attacks that exploit IT infrastructure vulnerabilities resulting from misconfigurations of network components, such as firewalls, overwhelming digital services by flooding requests (denial of service (DoS), DDoS) [31,34], software bugs in the system (such as structured query language (SQL) injections [30], privilege escalation [2], man-in-the-middle (MITM) or eavesdropping [42], Cyrptograhic Attack [30]; (ii) ransomware [20,32,42,53,53] attacks being launched against healthcare organisations, with the intention of causing service disruption and holding the healthcare organisation data hostage for economic gains; (iii) the emerging threat of exploiting human vulnerability in gaining access to healthcare infrastructure.

The history of cyber threats against IT systems can be traced back to 1982, with the first computer virus being released in the wild by a high-school student, called “Elk Cloner”, which was a harmless program that displayed a poem on Apple II computers [53]. Since then, over the last two decades, there have been several sophisticated developments in the design and development of cyberattacks [38]. The changing nature of cyber attacks against healthcare and clinical environments is also reflected by the scale and volume of digital strategies adopted within healthcare organisations.

A brief historical record of the different events that shaped the field of cybersecurity is presented in [53]. Since the first reported case of a virus developed in 1982, a total of 14 incidents have been documented, which resulted in the development of the U.S. National Institute of Standards and Technology (NIST) to publish the first version of “Framework for Improving Critical Infrastructure Cybersecurity” in 2014. In [30], the authors state that about a total of 94% of healthcare organisations globally have experienced data breaches of patient records, encountered information loss, been hacked or had their data displaced. The total volume of such cyber incident accounts to 150 million patient health records being breached between 2009 and 2014 in the U.S. During this period, it is also evident that an increasing number of healthcare organisations shifted their operations to adopt digital technologies, where patient records are maintained in digital format, thus providing an ideal platform for cyber attackers to launch systematic attacks against healthcare organisations [53]. While the popular opinion states that such data breaches should be attributed to external hackers, the research in [54] notes that most breaches are the result of employee carelessness and/or failure to comply with information security policies and procedures. Following the number of cyber incidents, there is increasing awareness about the need to protect patient data and electronic health records against external and internal attacks as noted in [51]. The authors promote the role of international standards such as ISO/IEC 80001-1, which provides a framework to bring together actors in cyber defence to enable enhanced clinical care delivery.

In contrast to the traditional cybersecurity attacks launched against IT systems and services of healthcare organisations, a major incident of ransomware attack was reported in 2016 when the Hollywood Presbyterian Medical Centre in Los Angeles paid a total ransom of USD 17,000 to hackers [53]. The incident set the precedence for launching attacks, which offer incentives and are motivated by financial gain, resulting in the disruption of healthcare service delivery. To create awareness about the impact of ransomware, [23] conducted a tabletop exercise to C-Level health care executives in a hypothetical scenario to develop strategies as countermeasures. The severity of ransomware threats was subsequently further analysed in [15], in which best practice recommendations were advised to enhance the cyber hygiene practice for healthcare physicians. In [6], the authors argued that to enhance cybersecurity in hospitals, the high-level management team comprising chief information officers and chief security officers should focus on enhancing stakeholder alignment in devising cybersecurity strategies. In contrast to the above cyberattacks, another form of threat has emerged, targeting healthcare professionals. Such threats, commonly categorised as “social engineering”, allow the attacker to exploit the large amount of public information hosted on social media platforms to gather the personal information of healthcare professionals [15]. Social engineering attacks are designed to circumvent traditional cybersecurity measures [18]. A well-known social engineering method is phishing. The role and impact of phishing attacks have been well studied in the literature [8,9,10,13,15,16,17,18,41,43,55,56].

In [55], the authors present a review on the state of cybersecurity in healthcare in which they consider cybersecurity breaches to include information stealing, ransomware attacks on hospitals and attacks on medical devices. Additionally, the authors also categorise the insider attacks to include the accidental or deliberate actions of healthcare staff that would compromise the cyber defence integrity of healthcare organisations. Such actions include responding to phishing emails, by which attackers can extract login credentials to infect the IT infrastructure with malware. Other human actions, acknowledged by the authors, include erroneous security settings, misuse of passwords and property loss (laptops with confidential data).

While there is a strong consensus among healthcare organisations to enhance the quality of service, there are economic barriers that prevent organisations from adopting cyber defence solutions. Such an issue was tackled in [8]. The authors argued that cybersecurity measures are not only a source of expense, but rather a chance for value creation. The authors introduced the term “value-based healthcare (VBH)” in which they aim to link the reimbursement of health care from insurance organisations to quality of healthcare services. One of the instances noted in the article cites the business service disruption caused across the U.K., due to the WannaCry cyberattack, which caused the National Health Service (NHS) to cancel nearly 600 surgeries and more than 19,000 appointments [56]. While the direct cost of such attacks is quantified by the ransom amount paid out to the malicious attackers, the indirect cost to the organisation is measured by reputation loss and negative impact on its service delivery. Such qualitative measures should also be considered upon considering the cost of investment in building cyber defence capabilities with healthcare organisations [57].

Following the increasing reports of phishing attacks launched against healthcare professionals, research in [13] reported the root cause of employees of an organisation still clicking on phishing links. The analysis was presented in the context of theory of planned behaviour (TPB) and integrating trust theories. The authors concluded that, from an analysis of attitude, subjective norms, and perceived behavioural control, there is a gap between the decision-making process and the observed compliance behaviour. The authors observe that there is less intention for organisational compliance with cyber defence policies in comparison to the intention of clicking on phishing links [13]. Extending the impact of phishing attacks at a national scale, research in [9,10] reported on the effectiveness of a training program offered in the U.S. healthcare system against the phishing click rate. The authors observed that phishing is a common threat vector and within the simulated environment, employee click rates decrease with repeated simulation. In [15], the authors concluded that healthcare professionals have limited awareness of threats posed by social engineering attacks, particularly by phishing attacks, and there is a need to promote cyber hygiene and information governance policies among the healthcare professionals.

Addressing the cognitive load in processing emails by healthcare professionals, ref. [16] presents a framework for evaluating the amount of attention required in deciding whether an email is authentic or phishing. The authors conclude that there is not a systematic understanding of the psychological components of these attacks, resulting in their high success rate. In [43], the authors argue that cyber defence is a collaborative effort between employees and the administrative members of the healthcare organisation. As a result, the authors present recommendations aiding healthcare professionals to successfully identify phishing email attacks. In [17], the authors state that, in complement to the technological counterpart, there is a need to bring together organisational scientists to model human behaviour in reacting to phishing attacks.

### 3.2. Organisational Strategies to Strengthen Cyber Security Capabilities

Healthcare data breaches are identified as a growing threat to the healthcare industry, causing not only data loss and monetary theft, but also attacks on medical devices and infrastructure, threatening human lives [6]. The increasing frequency and the evolving nature of cyber attacks launched against healthcare and clinical environments requires an organisation-wide effort to undertake risk prevention and mitigation actions.

However, it is noted that several healthcare organisations are muddling through cybersecurity measures, due to the size and complexity of operations, coupled with the presence of numerous legacy and stand-alone systems. This is cited as the main difficulty in implementing effective cybersecurity measures [31]. The authors noted that the healthcare organisations have adopted the approach, “if it ain’t-broke-don’t fix it” among the senior healthcare management. The complexity of healthcare organisations were outlined in [6] in which the authors stated that the healthcare organisations are technology saturated environments, similar to other organisations. The hospital environment struggles to manage an array of devices ranging from legacy IT systems to more modern connected medical devices. Additionally, the internal policies of hospital organisations requires coordination among finance, IT, and human resources for effective administration, while offering support for specialised services, such as radiology, cardiology and paediatrics, among others.

The degree of specialisation required in each of these healthcare service delivery is unique and often requires totally different equipment to cater to the needs of different patients. These complex systems have different workflows and employ a highly specialised labour force that requires years to train. In contrast to other industrial sectors, healthcare organisations often are subjected to regulatory pressures imposed by governmental agencies and regulatory authorities. The security of personally identifiable information (PII) is of paramount importance within healthcare. PII is characterised by any data that could potentially be used to identify a particular person. Examples of such information include a full name, social security number, license number, bank account number, passport number, and email address, among others.

Finally, the authors also argue that healthcare organisations are driven by patient-centred care in which patient services take precedence against revenue generation. The financial limitation of healthcare organisations has been identified across several studies in the literature [6,17,30,31,52].

In [48], the authors argued that the cyber domain is a multi-disciplinary field bringing together expertise from computer science, mathematics, economics, law, psychology and engineering. The observation further extends the study to include not only being limited among the interactions between networks of online devices, but to identify how humans interact and are influenced by these devices. The governance of such interactions is critical to be standardised across healthcare organisations to promote interoperability and regulatory compliance [51]. In the context of organisational strategies adopted to counteract cyberattacks, it is recommended that healthcare organisations create a specialised cyber security workforce framework [48]. The authors identified seven such roles within the framework, namely (i) security provision, whose responsibility is to conceptualise, design and build secure ICT systems; (ii) operation and maintenance, whose responsibility is to provide support, administration and maintenance of ICT systems; (iii) overseeing and governance, whose responsibility is to provide leadership, management and direction in implementing cybersecurity defence and resilience strategies; (iv) protection and defence, whose responsibility is to identify, analyse and mitigate threats to internal ICT systems and/or networks; (v) analysis, whose responsibility is to perform highly specialised review and evaluate the incoming cybersecurity information to determine its usefulness for intelligence; (vi) collection and operation, whose responsibility is to provide specialised denial and deception operations and collect cybersecurity information that maybe useful to develop intelligence; and (vii) investigation, whose responsibility is to investigate cybersecurity events or crimes related to ICT systems, networks and digital evidence. While these organisational roles to be created adopt a bottom-up approach in which the information is propagated from the operational field to the management, in [6], the authors argued that cybersecurity strategies should adopt a top-down approach, with chief information officers (CIOs) and chief information security officers (CSIOs) setting up the vision for enhancing organisations’ cyber resilience. In complement of the observations of [48], the authors in [6] stated that cybersecurity capabilities encompass a variety of programmes, behaviours and technologies that a hospital employs to improve cyber resilience. However, some of these strategies are not self-sustaining and erode over time [6,48].

The literature review on organisational strategies could be broadly classified into two main categories: (i) technical solutions adopted to enhance cyber resilience, and (ii) human factor approaches to strengthen cyber defence. In the context of the technical approach reported in the literature, the adoption of organisational strategies relates to collaborative information sharing systems in which a cyber attack incident is shared with the rest of healthcare organisations [38]. In this article, the authors argue that information collected during cybersecurity incidents should be shared with other healthcare organisations for blacklisting the incident origins. The information exchanged between the organisations could encompass data, such as IP addresses, the communication connected between internet protocol (IP) addresses, and the interactions and browsing patterns of users. The recipient of this information could swiftly reconfigure the IT systems and update rules on the firewall to prevent a similar attack. The authors propose the use of standard information exchange, such as Threat Information Expression (STIX) [58] and Trusted Automated Exchange of Intelligence Information (TAXII) [59] to communicate cyber incidents. In complement to sharing the information on cyber incidents, the authors in [18] recommended the use of international standards ISO/IEC 27002:2013 [60] and ISO 27799:2016 [61] for enhancing cyber resilience within healthcare organisations. The ISO/IEC 27002:2013 standard offers guidelines on the management practices to be adopted, considering the risk profile of the environment. The standard promotes the usage of industry accepted information security controls to protect the ICT infrastructure. On the other hand, the ISO 27799:2016 standard on “health informatics” offers guidelines for securing the organisational ICT infrastructure based on recommended management practices. Additionally, the range of technical mitigation measures identified include (i) regular backups, as recommended by ENISA [62] guidelines; (ii) implementing firewall and network segmentation; (iii) disabling unused physical ports for blocking access to universal serial buses (USBs); (iv) white listing permitted applications; (v) the adoption of least privilege principle for managing user authentication and access rights to healthcare resources; (vi) performing regular updates and patches; (vii) implementing software programs for virus and malware protection; (viii) adopting the encryption of data both at rest and in transit; (ix) implementing audit trail and logging for incident reports; (x) implementing the application for network monitoring and intrusion detection; (xi) secure system configurations; and (xii) protecting mobile devices for Bring-Your-Own-Device (BYOD) services. The importance of system configuration is highlighted in [43], along with ensuring the implementation of reliable system defence based on implementing user-focused strategies.

In complement to the technical solutions for mitigating cyber attacks, the organisational strategies developed based on human factors reported in the literature include [9,17,20,25,30,43,54]. A comprehensive analysis is presented in Section 3.4.

### 3.3. Cyber Risk Assessment Methodology within Healthcare Organisations

The impact of digital technologies and the adoption of digitalisation strategies has enabled healthcare organisations to deliver teleconsultation and tele-expertise, store patient records in an electronic format and interface with connected health devices [33]. The pervasive nature of digital technologies requires a detailed analysis of the different risk assessment methodologies being adopted within healthcare organisations. According to ISO/IEC 27000:2018 standard [63], information security is defined as the measure of confidentiality preserved along with the integrity and availability of data processed by computer systems. However, in a complex organisation of healthcare systems, a broader terminology should be adopted [33]. Historically, threats to healthcare organisations were classified into physical, such as fire or power interruption, unauthorised physical or electronic access, and authorised physical or electronic access [64]. Since the publication of such a categorisation of risk assessment, the nature of threats have evolved; currently, the challenges faced by healthcare organisations are related to cyber attacks. In the development of a risk-assessment methodology, authors in [33] argued that it is vital to model different forms of threat. They stated that threat intelligence should be built on evidence-based knowledge, which includes context, mechanisms, indicators, implications and actionable advice on the emerging menace or hazard to assets. In the literature, there have been several attempts reported on formalising the threat modelling methodologies and threat classification models, such as Structured Threat Information eXpression (STIX), Operationally Critical Threat, Asset, and Vulnerability Evaluation (OCTAVE), Spoofing, Tampering, Repudiation, Information Disclosure, Denial of Service, Elevation of Privilege (STRIDE), the Trike conceptual framework for security auditing, and Visual, Agile, and Simple Threat Modelling (VAST) [33,64]. In [64], a detailed analysis of the threat modelling process to be adopted for the development of cyber risk assessment is presented.

In [65], the authors identified the additional risk introduced by integrating connected medical devices and offered recommendations on organisational policies to be adopted for conducting risk assessment. The authors recommended the use of international standards for documenting, change management, risk management and responsibility assignment to be addressed from a management perspective, e.g., ISO/IEC 80001 standard, “Application of risk management for IT-networks incorporating medical devices - Part 1: Roles, responsibilities and activities”, from the International Standard Organisation (ISO) and the International Electrotechnical Commission (IEC). A similar case study was presented in [7], in which the authors promoted the need for developing methodologies to protect information resources from sophisticated and persistent cyberattacks from both scientific and practical perspectives. The investment from an organisation into formal controls (e.g., risk management, policy and procedures), informal controls (e.g., training), technology controls (e.g., firewalls, intrusion detection systems, anti-virus software, and layers of encryption), physical controls, administrative control (e.g., Control Objectives for Information Technologies (COBIT), ISO/EIC 27001, NIST 800-53). Regulation (EU) 2016/679, General Data Protection Regulation (GDPR), the Health Insurance Portability and Accountability Act of 1996 (HIPAA), Public Law 104-191 of the U.S. Government, and the Sarbanes–Oxley Act (SOX) federal law of the United States of America protect investors from fraudulent financial reporting by corporations and the Payment Card Industry Data Security Standard (PCI-DSS).

In [54], the authors stated that the majority of data breaches lies with employee negligence and/or carelessness surrounding information security, something that cannot be fully mended through legislative or technological remediation. As a result, the author argued that the cybersecurity risk methodology should focus on modelling employee behaviour surrounding information security. The authors introduced the Information Security Climate Index (ISCI), a parsimonious (that includes nine items) tool that was developed with two pilot studies, representing an extensive validation effort based on best practices in scale development. Additionally, in [31], the authors stated that managing cybersecurity risk is a balancing act between security and resilience. The authors proposed a three stage cybersecurity risk management road map with (i) understanding cybersecurity risks; (ii) valuing cybersecurity risks and mitigation measures; and (iii) communicating cybersecurity actions and solutions. The risk assessment methodology includes the identification of core, mission-critical functions and processes to develop an inventory of vulnerable assets associated with the core functions and processes. The methodology allows for the assignment of a risk impact score to each vulnerable asset. In [52], the authors presented the case study of the WannaCry attack on the U.K. NHS services, which negatively impacted healthcare service delivery. The reasons attributed in the article refer to the lack of ability to adopt to evolving technological challenges [52].

As noted in Section 3.1, one of the evolving threats to healthcare services is “social engineering” attacks. In this context, several articles have been published to evaluate the response of organisations to promote cyber hygiene among healthcare professionals and relevant stakeholders [7,17,25,40,41,43,48,49]. In each study, an overview of the risk assessment methodology that relates to the vulnerability of individual assets was evaluated. The authors acknowledged that, while the existing practices have focused on the technical development to counteract cyber security measures, the evolving nature of cyber threats requires an insightful analysis on human behaviour. The authors remarked on the need to develop organisational strategies to promote cyber threat awareness and offer training to healthcare professionals to follow organisational guidelines on cyber hygiene practices.

### 3.4. Role of Human Factors in Improving Cyber Resilience

The role of human factors in improving cyber resilience has been reported in 21 articles as outlined in Table A2. The 21 articles were subjectively ranked by the authors with individual scores being assigned to each publication. In this section, we present a summary analysis of the key aspects being addressed in the literature that relate to the impact of “social engineering” attacks launched against healthcare professionals. The publications are classified into three main categories, namely (i) training and awareness activities on social engineering attacks (e.g., phishing) [8,9,10,11,12,13,14,15,16,17]; (ii) activities related to promoting general awareness on information security against cyber attacks [18,19,20,21,22,23]; and (iii) best practice recommendations adopted in promoting cyber hygiene from other industrial sectors [24,25,26,27,28,66].

To the best of our knowledge, one of the early efforts reported in the literature on modelling the role of behaviour training in healthcare was published in [11]. The primary focus of the study was to strengthen the organisational defence against cyber attacks. The goal was accomplished through the creation of a training program that focused on employee habits. The authors adopted the use of the Martin–Morich model of consumer behaviour, which was later extended to study information security among healthcare employees. The rationale presented in the publication reflected upon the human nature of performing repetitive tasks—The behaviour rapidly becomes automatic and does not require conscious control. The authors observed that despite the increasing number of governance regulations requiring a high degree of compliance to protect against cyber attacks, the automatic behaviour of healthcare employees results in low habitual practice [11]. To overcome such challenges, the authors presented the scope of the training programme delivered to the healthcare professionals. The training program focused on three types of common cyber attacks, namely phishing, password sharing and cloud service. The authors concluded that habits are more powerful than other types of thoughts; thus, to enhance the cyber resilience of a healthcare organisation, it is vital to monitor and deliver habit-changing training policies. Since the first report, there have been several subsequent studies that were conducted to improve awareness of cybersecurity [7,11,22,34].

As noted in Section 3.1, the latest form of social engineering attacks that is causing data breaches is phishing attacks [9,10,13,14,15,45]. Despite organisational efforts to promote the threat of phishing attacks for the healthcare sector, hospitals still significantly suffer from such attacks, impacting the quality of care and safety of patients. To study the rationale behind such a schema, the authors in [13] conducted a case study to investigate why hospital employees decide to click on phishing emails by analysing the actual data. The authors conducted the study in compliance with the theory of planned behaviour (TPB) along with integrating trust theories. The study results indicated a positive correlation between the workload and the click rate on phishing links. The study concluded with recommendations for the healthcare organisations to develop strategies for managing a balanced workload of hospital employees to improve cyber resilience. Similarly, in another study presented in [15], the authors emphasised the need to maintain “cyber hygiene” and information governance through mandatory training programmes, which resulted in an increased understanding of these risks. The study also concluded that, while many healthcare professionals are aware of phishing attacks and respond appropriately, ongoing education is required across the spectrum of cybersecurity, with special emphasis around the “leakage” of information on social media.

In [9], the authors presented the results of a phishing campaign conducted across six geographically dispersed U.S. healthcare institutions that ran phishing simulations from 2011 to 2018. The phishing attacks launched were classified into three categories, namely (i) office related, (ii) personal, and (iii) ICT related. The study concluded with the observation that in the beginning, one in every seven phishing links were clicked on by the employees; however, with the increasing number of training programmes, the rate has fallen down, suggesting the potential benefit of the simulation and awareness programmes. Complementing the earlier studies, in [16], the authors argued that social engineering attacks are a kind of psychological attack that exploits weaknesses in human cognitive functions. Therefore, as a countermeasure, the authors stated a deeper understanding of which aspects of human cognition are exploited by these cyberattacks and why humans are susceptible to these cyberattacks. The study concluded by formulating the short-term cognition factors that affect the human ability to make right decisions. These short-term cognition factors include (i) employee workload, (ii) stress, and (iii) vigilance. In contrast, the long-term cognition factors include (i) personality, (ii) expertise, (iii) individual differences, such as gender and age, and (iv) culture. In one of the large-scale field experiments conducted to evaluate the effectiveness of the awareness and training programme, a case study was reported in [14]. In this study, the authors evaluated the benefits of informing, simulating or both to evaluate the click rate of phishing links by healthcare employees. The study concluded with a series of observations that (i) personal experience was more effective in threat identification; (ii) information shared through email on the threat of phishing had limited impact, as the authors could not evaluate whether the recipients had read the relevant literature; (iii) infographics were useful to promote awareness; and (iv) the combination of simulation and information had less impact.

The observations reported in the literature on the importance of promoting awareness among healthcare professionals are reflected across the healthcare industry, with the general consensus being formed on the necessary use of integrated, mature, secure and safe digital technologies to offer health services and maintain the quality of care [8]. The authors also echo that technologies alone have no value in themselves and cannot guarantee the expected value if the organisation culture and internal environment does not provide leadership on governance and compliance. The authors consider the high level of interest in using personal devices to access healthcare records and data under the BYOD scheme to be causes of concern among healthcare professionals, who are unaware of the threats of cybersecurity. While the authors acknowledge the delivery of training programmes to healthcare professionals, they consider the training programmes to be universal and not tailored to suit the needs and demands of the healthcare industry.

As noted earlier, the need for cybersecurity training among healthcare professionals has been addressed in other studies in which the authors do not envisage the threat of phishing. In [18], the authors proposed a recommendation on offering user training and simulations to raise awareness and training on IT systems related to cybersecurity. The authors also distinguished between the awareness-raising and training activities based on the target audience. On the one hand, awareness raising activities are targeted at individuals who are able to identify potential security risks and respond appropriately. On the other hand, training programmes are formal and have the goal of building knowledge and skills in cybersecurity measures. In [19], the authors presented a study on the stakeholder perception of categorising connected medical devices in which IT personnel and medical technology members participated to share their views on the cyber security risks posed by respective devices. The study concluded that segmentation of medical devices based on the risk assessment would increase cyber security measures, but would also increase administration and resource needs.

In another study presented in [21], the authors aimed to evaluate the participants’ perception about security threats, effectiveness and costs of safeguards, self-efficacy, susceptibility, severity and their motivation and actions to secure their mobile devices. The study concluded that healthcare professionals perceive the severity of threat to their mobile data but do not feel personally vulnerable [21]. The authors acknowledged that the participants were knowledgeable about security safeguards but their knowledge of the costs and problems related to the adoption of these measures was mixed. The study concluded by highlighting the necessity to increase security awareness among the healthcare professionals through targeted programs. In [22], the authors conducted a study to evaluate the awareness of healthcare professionals on the medical profession, computer proficiency, experience and their place of employment. The authors concluded the study with an observation on the reduced awareness of healthcare professionals on information security. In a further study reported in [23], the authors developed and facilitated a tabletop cybersecurity simulation exercise aimed at management executives across healthcare organisations. The exercise was attended by more than 70 members, representing 11 countries. The authors concluded the study by highlighting the experiences of the participants, which highlights the lack of institutional attention offered to cybersecurity as opposed to epidemics or natural disasters.

Cybersecurity training has been an active area of investigation for the past several years, even beyond the scope of the healthcare industry. One of the seminal works recently published on the curriculum development for cybersecurity was reported in [24]. The authors proposed curriculum development based on the combination of practical hands-on challenges prepared by security experts and formal study programs facilitated by professional educators. In [25], the authors reported on the development of threat perception among employees to positively influence threat appraisals and coping appraisals as mediators in the information security area. Following the need to deliver training programs to improve the knowledgeable skills of the organisation employees, ref. [28] proposed a training program methodology in which participants are active and offered actionable insights relating to cyber awareness by employing the lessons learned in their personal life. The authors stated that the “relaxed alert” state of employees was designed to minimise security fatigue for employees. In [27], the authors observed that, while several articles in the literature propose to offer training programmes to employees, they fail to state the frequency of such training programmes and the need to update the content of such program delivery. The authors acknowledged the changing patterns of cyber attacks being launched against organisations, and referred to the use of open data sets, such as Linked Open Data Databases and DBPedia, to offer training to employees.

### 3.5. National Studies on Cybersecurity Ecosystem within Healthcare

Complementing the organisational cybersecurity measures, several articles also reported on the national strategies being adopted [8,9,10,22,31,50,51,52]. National case studies reported from the U.S. dominate the literature, with supplemental studies reported from Canada (1), Denmark (1), Romania (2) and the U.K. (4).

## 4. Discussion

This systematic review is motivated by the need to summarise the literature on the role of human factors for cybersecurity within the healthcare sector. A total of five research questions were identified to guide the collection of articles from the literature. Following an extensive analysis of 695 articles, a final list of 70 articles was selected for this study.

Besides answering the research questions identified, during the early stage of this paper, the objective of our systematic review was to collect evidence on the evolving nature of cyber threats faced by healthcare organisations and clinical environments, who store patient records and personally identifiable information. The scope of cyber attacks is broadly classified into three categories: (i) attacks against the IT infrastructure for *service disruption*; (ii) attacks launched for *personal economic gains*; and (iii) social engineering attacks targeting healthcare professionals. The classification of the different attack vectors also highlight the evolving nature of cyber attacks, which are increasingly relying on exploiting healthcare professionals’ personal information shared through social media platforms. The information collected through publicly accessible resources is used to launch phishing attacks against targeted individuals.

The high susceptibility rate of healthcare professionals and the failure to recognise phishing attacks are attributed to the high stress environments often encountered in hospitals. Additionally, the literature considers how the lack of sufficient training and awareness impacts healthcare organisations facing ever-increasing cyber threats. In the literature, authors have acknowledged that each healthcare organisation is unique and often faced with economic pressure to deliver patient care, with IT system security not being prioritised above medical services.

The lack of organisational roles, such as those of chief information officer and chief security information officer, has also been cited as a reason for the increasing number of data breaches reported across national healthcare infrastructures. Following the nationwide launch of ransomware attacks against the U.K. National Health Service (NHS) in 2016, there has been a visible increase in organisational defence strategies being adopted against cyber attacks. The changes in organisational policies resulting as a response to new forms of economic attacks against healthcare organisations include training programmes for professionals, according to laws and regulations, the impact of data security, and the need to cultivate the habit of cyber hygiene. Several healthcare organisations promote cybersecurity awareness among their employees, with participants ranging from nurses, doctors, nurses, admin personnel and the management team. In addition to the training programmes and awareness campaigns, some organisations have conducted studies on the “click rate for phishing attacks” by simulating social engineering. The results from such studies identified the high degree of reliance on technology to successfully flag phishing emails; those that were not flagged were considered to be genuine communications. The increasing degree of training and resultant awareness in healthcare professionals is proven crucial to combat phishing to a very large extent.

In the literature, the impact of social engineering is identified as a key motivating factor to secure patient information. The use of information from social media has allowed cyber attackers to launch personalised and targeted attacks against healthcare professionals. Such a phenomenon has given rise to attempts to achieve compliance through cyber hygiene practices for healthcare professionals. In addition to security training being offered to healthcare professionals, the security and privacy settings from social media platforms (such as Twitter, Facebook and LinkedIn) should be promoted. In order to address security gaps within healthcare, each organisation should perform a cyber risk and privacy impact assessment and identify potential vulnerabilities, which could be exploited by cyber actors.

The formal specification of risk assessment methodologies reported in the literature is often limited, which could be attributed to the limited amount of sensitive information published in the public domain. Nevertheless, a broad set of approaches to strengthen countermeasures against cyber threats are proposed in the literature. The selected articles in this systematic review highlight the need for timely software and hardware updates, improved security protocols being adopted for ICT technologies (such as password rotation policies, restrictions on password structure, and many others). The vulnerabilities introduced from the exchange of information among healthcare professionals has also been identified as a source of cyber threat. Lastly, the vulnerabilities introduced from the adoption of the connected infrastructure of medical devices has been studied in the literature.

While several organisations and researchers have identified the need for delivering cybersecurity training to healthcare professionals, there is no consensus within the community about the mode of delivery, the curriculum of the training programme and training assessment criteria. Following the classification of healthcare as a critical infrastructure, articles related to data governance, regulatory compliance for technical standards, such as ISO/IEC 80001-1 [51], have not been effectively translated into formalising methodologies for strengthening human factors. The evidence reported in the literature review presents organisational effort in developing cyber resilience strategy, while the concerted effort across organisations and within the healthcare industry is still lacking. The limited number of case studies, which have been carried out within organisations, have concluded the need to develop strategies for healthcare professionals to play a central role in mitigating cyber risks.

Our research findings highlight the need to establish formal training and educational standards to empower organisations to address human factors of cybersecurity, critically mitigating cyber risks. Despite the increasing number of research articles, we consider the research on human factors to still be in its infancy within healthcare industries, which is supported by the fragmented case studies and research outcomes presented. To launch a coordinated effort on promoting good practices on cybersecurity measures, we need to adopt an approach where IT systems are able to detect phishing emails and other social engineering attacks, while at the same time, equip healthcare professionals with the knowledge to recognise social engineering.

## 5. Conclusions and Future Work

The systematic review presented in this article was designed to study the current literature on the role of humans in strengthening cyber security defences. A total of 70 articles were selected to be included in the review from a total of 695. Best practices and the recommendations provided by healthcare organisational experts should be promoted among healthcare stakeholders, including doctors, nurses, patients, administrators, and IT personnel. Despite researchers having studied the role of human factors, there is a critical need to develop a systematic methodology to harmonise the research findings that could be objectively evaluated by cybersecurity experts in the context of securing the IT infrastructure of healthcare industry. Future work on evaluating the effectiveness of training and awareness campaigns in healthcare will benefit from investigating different threat techniques and scenarios. There is also a need to develop objective metrics for unifying national studies to promote cyber hygiene across healthcare. From our systematic review, we conclude that a collaborative and standardised approach for the development of training programmes, awareness campaigns, and information sharing on the nature and type of cybersecurity attacks is required to collectively strengthen healthcare organisations against ever increasing cyber threats.

## Figures and Tables

**Table 1:** Research questions and background considered for investigation in this systematic review.

Background	Research Questions
There are several types of *cyber attacks* launched against healthcare organisations [30]; the most successful ones target healthcare professionals using *social engineering methods*, leveraging personal data shared through social media [3]	**RQ1**: What are the most common types of *social attacks* against humans encountered by healthcare organisations?
*Organisational resilience* against cybersecurity relies on robust data governance policies extending from data security, privacy and IT infrastructure security among others [7,31]. While these strategies have offered protection against traditional cyberattacks (e.g., Distributed Denial of Service (DDoS)), the emergence of threats, such as *ransomware* (e.g., WannaCry) attacks have shown a tremendous increase in the frequency of data breaches in healthcare organisations [20,32].	**RQ2**: What *policies and governance* adopted by healthcare organisations have resulted in enhanced resilience?
The effectiveness of cybersecurity measures are positively associated with *cyber risk assessment methodologies* adopted within healthcare organisations [33]. The growing complexity of healthcare services offered to patients along with the use of digital technologies, provide an ideal platform to data breaches. While traditional risk assessment studies have focused on evaluating the security profile of IT systems, the emergence of social engineering dictates the need to review the healthcare *cyber risks caused by insecure human behaviour*.	**RQ3**: How does an organisation perform *cybersecurity risk assessment* with regards to identifying the role of humans in the loop for enhancing cybersecurity?
*Cybersecurity education and training* are increasingly becoming a part of cyber defences adopted by healthcare organisations [9,34,35], for example, training to detect phishing emails. Following reports on the use of information collected from social media to launch targeted cyber attacks against healthcare professionals, it is important to promote awareness on such an evolving nature of threats [22].	**RQ4**: What is the role of the *training programme* in enhancing healthcare professionals’ awareness about cyber threats, and how can we measure the impact of *training and awareness activities* adopted within an organisation?
Healthcare is a *critical infrastructure* across Europe and globally [33], and its service disruption can lead to a national emergency. International standards organisations (such as the European Union Agency for Cybersecurity (ENISA)) offer recommendations for enhancing *national cyber resilience* [18]. Coordinated cyber attacks launched across nations result in both economic and human loss.	**RQ5**: What *cyber defence strategies* have been proposed by national and international organisations to strengthen cyber resilience?

## Appendix A

A summary of the terms used in the review are outlined in Table A1.

**Table A1:** Glossary terms [30] used in the systematic review.

Term	Definition
Cryptographic attack	An attack carried out with the intention of revealing information that has been concealed
Cyber attack	The act of intentionally disrupting data information
Cyber defence	The ability to prevent cyber attacks from infecting a computer system or device
Cyber resilience	The ability of an organisation to continue delivering healthcare services to patients despite adverse cyber events
Cyber risk	Exposure to harm or loss resulting from breaches of or attacks on information systems
Cyber threat	The possibility of a malicious attempt to damage or disrupt a computer network or system
Data breach	This is when information is lost, stolen, displaced, hacked or communicated to unofficial recipients
Denial-of-service (DoS)	An attack that aims to flood a network with traffic in order to disrupt service and prevent users from accessing network resources
Distributed Denial-of-service (DDoS)	A malicious attempt to disrupt the normal traffic of a targeted server, service or network by overwhelming the target or its surrounding infrastructure with a flood of Internet traffic. DDoS attacks achieve effectiveness by utilizing multiple compromised computer systems as sources of attack traffic
Malicious software or Malware	A group of programs that are designed to harm or compromise a computer system without the permission of the user
Man-in-the-middle (MITM) or Eavesdropping	A reconnaissance attack in which an intruder intercepts communication between two parties. The attacker eavesdrops on the contents communicated by secretly acting as an intermediary in the information exchanged
Phishing	The use of social engineering to trick individuals or organisations into either divulging information or perform an activity harmful to their computer
Privilege Escalation	Attacks driven by the goal of achieving a higher level of access to a network or exploiting vulnerabilities in a program or network
Spyware	A software that is installed on a computer without the user’s knowledge, which transmits information about the user’s computer activities over the Internet
Ransomware	A type of malicious software designed to block access to a computer system until a sum of money is paid
Structured Query Language (SQL) Injections Exploit	Attack that exploit vulnerabilities in SQL to execute malicious “payloads" (harmful SQL statements), that make the data servers divulge information
Trojans	A type of malware designed to appear as useful, legitimate software
Virun	A common malware that self-propagates without the permission of the user and infects other computers
Worms	A type of malware that does not rely on a host file to run, self-replicate or propagate

## Appendix B

In the following table, a detailed review of the consolidated rankings associated to each publication included in the systematic review is presented. The individual rankings are offered as a guide to the reader to further explore the scope of research outlined in the literature.

**Table A2:** Summary of articles included in the review, searched in compliance with PRISMA recommendations.

S. No.	Publication Title	Publication Matching Score				
		**RQ1**	**RQ2**	**RQ3**	**RQ4**	**RQ5**
1	Sharing is Caring: A collaborative framework for sharing security alerts [38]	1	10	5	0	0
2	Cybersecurity Challenges for PACS and Medical Imaging [18]	10	10	10	8	0
3	Transforming Healthcare Cybersecurity from Reactive to Proactive: Current Status and Future Recommendations [30]	10	10	10	10	0
4	Information security climate and the assessment of information security risk among healthcare employees [54]	8	10	10	6	0
5	Information Technology and Medical Technology Personnel’s Perception Regarding Segmentation of Medical Devices: A Focus Group Study [19]	6	4	7	8	8
6	Proactive Role of Clinical Engineering in the Adoption of ISO/IEC 80001-1 within Healthcare Delivery Organization [51]	10	9	8	6	8
7	Cyber-attacks and threats for healthcare—a multi-layer thread analysis [33]	4	3	8	2	0
8	Digital health: Cybersecurity is a value creation lever, not only a source of expenditure [8]	9	6	6	10	8
9	Muddling through cybersecurity: Insights from the U.S. healthcare industry [31]	6	10	10	4	6
10	Evaluation of a mandatory phishing training program for high-risk employees in the U.S. healthcare system [10]	8	8	4	10	9
11	Intelligent and Dynamic Ransomware Spread Detection and Mitigation in Integrated Clinical Environments [42]	4	0	0	0	0
12	Where is the Risk? Analysis of Government Reported Patient Medical Data Breaches [67]	2	2	4	0	0
13	Security Assurance Against Cybercrime Ransomware [32]	6	4	4	0	0
14	Comprehensive user requirements engineering methodology for secure and interoperable health data exchange [68]	6	7	5	6	7
15	Cybersecurity in healthcare: A narrative review of trends, threats and ways forward [55]	8	6	7	8	8
16	Cybersecurity Education and Training in Hospitals Proactive Resilience Educational Framework (Prosilience EF) [34]	6	6	6	10	4
17	Conceptualizing the silent risk of inadvertent information leakages [69]	6	4	4	2	0
18	Cybersecurity: Role of Behavioural Training in Healthcare [11]	6	6	6	10	8
19	Can Cyberloafing and Internet Addiction Affect Organizational Information Security? [12]	1	1	0	8	9
20	Clinical Cybersecurity Training Through Novel High-Fidelity Simulations [35]	3	4	2	0	4
21	Cybersecurity Risks in a Pandemic [70]	6	7	8	0	9
22	Connected Medical Technology and Cybersecurity Informed Consent: A New Paradigm [71]	5	7	2	0	0
23	Cybersecurity in Hospitals: A Systematic, Organizational Perspective [6]	8	10	10	6	4
24	Maybe If We Turn It Off and Then Turn It Back On Again? Exploring Health Care Reform as a Means to Curb Cyber Attacks [56]	9	7	7	3	4
25	A cybersecurity primer for translational research [72]	6	7	6	3	3
26	Ethics and Phishing Experiments [44]	6	4	2	6	0
27	Why Employees (Still) Click on Phishing Links: Investigation in Hospitals [13]	8	8	6	10	2
28	Assessment of Employee Susceptibility to Phishing Attacks at U.S. Health Care Institutions [9]	10	4	4	10	10
29	Hacking the Human: The Prevalence Paradox in Cybersecurity [73]	6	0	0	4	0
30	Physician Practice Cybersecurity Threats: Ransomware [20]	8	8	6	10	2
31	The Economics of Cybersecurity [74]	2	1	2	0	8
32	Breaking the cyber-security dilemma: aligning security needs and removing vulnerabilities [75]	1	0	0	0	2
33	Mobile Device Security: Perspectives of Future Healthcare Workers [21]	4	0	0	6	0
34	Informing, simulating experience, or both: A field experiment on phishing risks [14]	8	6	8	10	9
35	The challenges of cybersecurity in health care: the UK National Health Service as a case study [52]	6	10	9	10	9
36	Quantifying Phishing Susceptibility for Detection and Behaviour Decisions [45]	7	0	0	4	0
37	Holding the Line: Events that Shaped Healthcare Cybersecurity [53]	10	0	0	0	0
38	Cybersecurity expert: medical devices have ’a long way to go’ [76]	0	4	2	6	2
39	Cybersecurity Standards Are Standing Up to the Bad Actors [77]	0	0	4	0	0
40	Phishing in healthcare organisations: threats, mitigation and approaches [15]	8	4	6	10	4
41	How can hospitals better protect the privacy of electronic medical records? Perspectives from staff members of health information management departments [78]	2	2	4	1	0
42	A Deterrence Approach to Regulate Nurses’ Compliance with Electronic Medical Records Privacy Policy [79]	2	2	0	4	0
43	Repurposing Case-Based Learning to a Conversational Agent for Healthcare Cybersecurity [39]	4	0	0	6	0
44	A Simple Assessment of Information Security Awareness in Hospital Staff Across Five Danish Regions [22]	6	0	0	8	9
45	The Future Cybersecurity Workforce: Going Beyond Technical Skills for Successful Cyber Performance [48]	6	6	9	2	0
46	Human Cognition Through the Lens of Social Engineering Cyberattacks [16]	8	4	6	10	3
47	Understanding Phishing Email Processing and Perceived Trustworthiness Through Eye Tracking [46]	3	2	1	2	0
48	Email phishing and signal detection: How persuasion principles and personality influence response patterns and accuracy [47]	2	4	2	5	0
49	Social Engineering Attacks During the COVID-19 Pandemic [3]	0	0	0	4	0
50	Organizational science and cybersecurity: abundant opportunities for research at the interface [17]	8	9	7	10	0
51	Healthcare Challenges in the Era of Cybersecurity [71]	0	0	0	0	4
52	A Socio-Technical Approach to Preventing, Mitigating, and Recovering from Ransomware Attacks [43]	10	9	9	7	10
53	A Comprehensive Cybersecurity Audit Model to Improve Cybersecurity Assurance: The CyberSecurity Audit Model (CSAM) [80]	4	10	6	4	2
54	A framework for competence development and assessment in hybrid cybersecurity exercises [49]	2	8	7	2	0
55	From awareness to influence: toward a model for improving employees’ security behaviour [40]	1	6	7	0	9
56	Connected Medical Technology and Cybersecurity Informed Consent: A New Paradigm [71]	0	0	8	1	0
57	The Security Challenges Emerging from the Technological Developments [81]	0	6	4	0	10
58	Cyber Place Management and Crime Prevention: The Effectiveness of Cybersecurity Awareness Training Against Phishing Attacks [41]	10	6	7	10	3
59	Cyber Security Awareness, Knowledge and Behaviour: A Comparative Study [82]	7	5	6	9	10
60	Cybersecurity Challenges and the Academic Health Center: An Interactive Tabletop Simulation for Executives [23]	10	4	6	10	0
61	Cybersecurity compliance behaviour: Exploring the influences of individual decision style and other antecedents [83]	2	4	2	2	2
62	Cybersecurity knowledge and skills taught in capture the flag challenges [24]	6	0	0	9	0
63	Enterprise cybersecurity training and awareness programs: Recommendations for success [28]	2	5	4	8	0
64	How can organizations develop situation awareness for incident response: A case study of management practice [7]	2	9	7	0	0
65	Information Security Awareness in Romanian Public Administration: An Exploratory Case Study [50]	2	4	4	4	10
66	Investigating the impact of cybersecurity policy awareness on employees’ cybersecurity behaviour [25]	2	7	8	9	0
67	The Utility of Information Security Training and Education on Cybersecurity [26]	2	6	4	10	0
68	Perceptions of Cybersecurity Readiness among Workgroup IT Managers [84]	0	2	4	2	4
69	Adaptive security awareness training using linked open data datasets [27]	4	2	1	10	4
70	Healthcare Cybersecurity Risk Management: Keys to an Effective Plan [65]	6	8	10	5	10
